# pyTWMR: transcriptome-wide Mendelian randomization in python

**DOI:** 10.1093/bioinformatics/btae505

**Published:** 2024-08-10

**Authors:** Sergey Oreshkov, Kaido Lepik, Federico Santoni

**Affiliations:** Endocrine, Diabetes and Metabolism Service, Centre Hospitalier Universitaire Vaudois (CHUV), Lausanne 1005, Switzerland; Faculty of Biology and Medicine, University of Lausanne, Lausanne 1005, Switzerland; Department of Computational Biology, University of Lausanne, Lausanne 1015, Switzerland; University Center for Primary Care and Public Health, University of Lausanne, Lausanne 1005, Switzerland; Swiss Institute of Bioinformatics, Lausanne 1015, Switzerland; Endocrine, Diabetes and Metabolism Service, Centre Hospitalier Universitaire Vaudois (CHUV), Lausanne 1005, Switzerland; Faculty of Biology and Medicine, University of Lausanne, Lausanne 1005, Switzerland; Institute for Genetic and Biomedical Research (IRGB) - CNR, Monserrato 09042, Italy

## Abstract

**Motivation:**

Mendelian randomization (MR) is a widely used approach to estimate causal effect of variation in gene expression on complex traits. Among several MR-based algorithms, transcriptome-wide summary statistics-based Mendelian Randomization approach (TWMR) enables the uses of multiple SNPs as instruments and multiple gene expression traits as exposures to facilitate causal inference in observational studies.

**Results:**

Here we present a Python-based implementation of TWMR and revTWMR. Our implementation offers GPU computational support for faster computations and robust computation mode resilient to highly correlated gene expressions and genetic variants

**Availability and implementation:**

pyTWMR is available at github.com/soreshkov/pyTWMR.

## 1 Introduction

Genome-wide association study (GWAS) is one of the major tools for the investigation of genotype–phenotype relationships in large cohorts (MacArthur *et al.* 2017). Known limitations of GWAS concern primarily the identification of the biological mechanism (i.e. the causal genes) from the associated regions in linkage disequilibrium with the causal variant (Tam *et al.* 2019).

Alternative approaches based on transcriptome-wide association studies (TWAS) are used to reveal links between genes and traits through gene expression. For a large cohort, for which genotypes, transcriptomics, and phenotypes are available, two-stage least-squares regression might be applied. However, (i) building such a cohort is very expensive, (ii) it is not straightforward to extract causality directions between genes and traits from regression analyses (Porcu *et al.* 2019, 2021).

To overcome these issues, Porcu *et al.* proposed a two-step Mendelian Randomization approach, transcriptome-wide summary statistics-based Mendelian Randomization approach (TWMR), that utilizes the genotypes/traits association and publicly available Expression quantitative trait loci (eQTL) datasets linking genotypes and gene expression. This highly cited approach belongs to the family of methods that seek for causal inference through Mendelian Randomization where the genetic variants are the instrumental variables, genes expression are the exposures, and the trait of interest is the outcome (Porcu *et al.* 2019). The same authors also proposed a “reverse” version (revTWMR) that combines trans-eQTLs with GWAS summary statistics to evaluate the effects of phenotypes on gene expression (Porcu *et al.* 2021).

Notably, the same approach can be used to explore the causality of effects for other omics such as proteomics and methylomics with the crescent availability of protein quantitative trait loci (pQTLs) and methylation quantitative trait loci (meQTLs).

In order to facilitate the use of TWMR and revTWMR, we re-implemented the original R scripts in an optimized and faster Python 3 package with the optional GPU support. We have also introduced modification to the original implementation to make them robust to highly correlated genetic variants and gene expressions.

## 2 Results

pyTWMR is a reimplementation of the original R scripts in Python3 with the support of NumPy and PyTorch for GPU processing. Our version reproduces the results obtained with the original scripts.

In TWMR, the causal effect of genes expression on trait is estimated with via the inverse-variance weighted method (Burgess *et al.* 2013):

(1) α^=E′C-1E-1E′C-1G

where *E* is a matrix of univariate effect sizes of SNPs on gene expressions (from available eQTL datasets), *G* is a vector containing the standardized effects of these SNPs on the trait of interest (from GWAS studies) and C is a linkage disequilibrium matrix for the same SNPs without any fully correlated genetic variant.

Significance levels (*P*-values) of causal effects for each gene is obtained through the Z-statistic α^SE(α^), with SEα^=var(α^)and the variance estimated with the Delta method (Ver Hoef, 2012 and Supplementary material) (mathematical derivation in Supplementary material):

(2) varα^=∂α^∂E2 * varE+∂α^∂G2 * varG+∂α^∂E*∂α^∂G * covE,G

where *cov(E, G)* is the covariance between effect sizes of SNPs on gene expression and trait and it is equal to 0 if these effect sizes come from different studies. For revTWMR, we implemented an equivalent approach for causal effects of traits on gene expression.

A possible complication is the computation of causal effects on highly correlated genetic variants and co-expressed genes. Multicollinearity inside computational matrices introduces numerical instability and numerical precision issues. In this case the matrix $E^{\prime }C^{-1}E$ in Equation (1) might be singular or near-singular, and the computation of the inverse would be numerically instable or even impossible. Here, we follow the same strategy proposed in the original article (Porcu *et al.* 2019) where independent SNPs and low correlated genes were preselected (*r*^2^<0.1 and *R*^2^<0.4, respectively).

**Figure 1.**
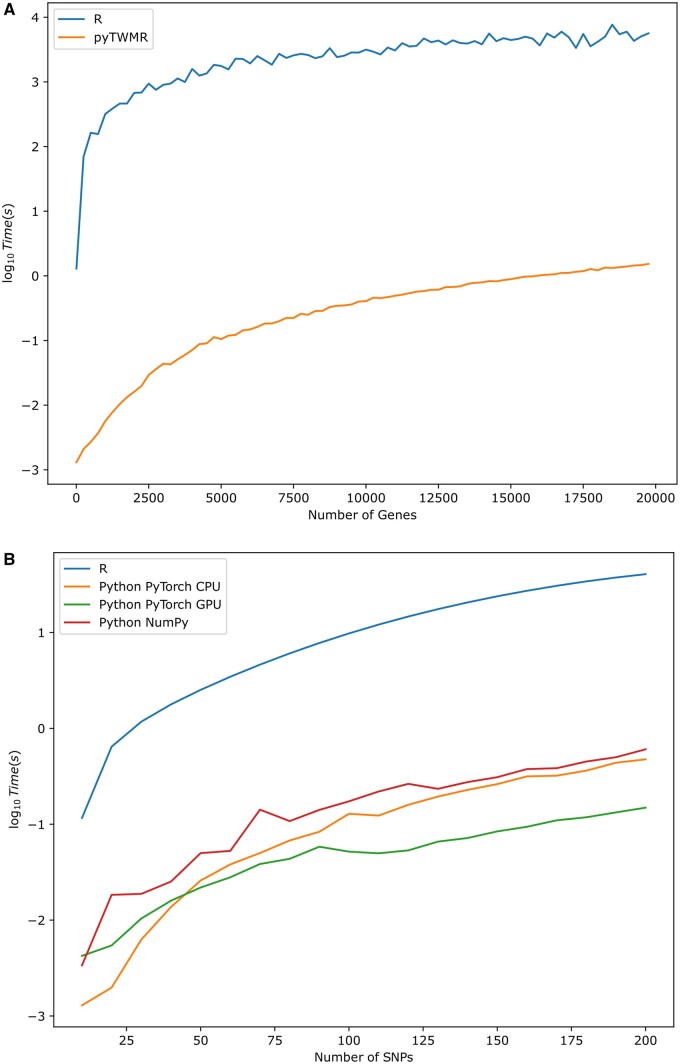
Scalability comparison. (A) Original R and our CPU-based implementations in function of the number of genes. (B) Average computation time in function of number of SNPs for original R, NumPy with LAPACK backend, PyTorch CPU mode based on OpenMP and PyTorch GPU mode with CUDA for nVidia GPUs. GPU is slower when processing small size input data due to initialization time cost and data transfer to GPU memory. Dataset from Porcu *et al.* (2019).

From the perspective of computational performance, CPU versions (both NumPy and pyTorch) are 40–50 times faster than the original R implementation, despite using similar underlying arithmetic computation algorithms (Fig. 1A). The GPU implementation offers 3–4 times increase versus CPU in terms of speed, especially in case of large number of genetic variants (Fig. 1B) enabling the computation of larger matrices. In our tests GPU implementation was able to compute more than 6000 genetic variants over more than 300 genes using nVidia Tesla A100 GPU. Moreover, it performed faster than its CPU counterpart while running on a laptop (Intel i7-10850H, 32 Gb RAM, GPU nVidia Quattro T2000).

## 3 Conclusions

We implemented a fast (GPU powered) and numerically robust version of the original TWMR approach to leverage transcriptome-wide data and GWAS studies to estimate the causal effects of genetic variation on complex traits. pyTWMR also integrates revTWMR which allows estimating trait-to-gene-expression causal effects.

## Supplementary Material

btae505_Supplementary_Data

## Data Availability

No new data were generated or analysed in support of this research.
