# Supplementary material for: pyTWMR: transcriptome-wide Mendelian randomization in python
Source: Bioinformatics. 2024 Aug 10;40(8):btae505. doi: 10.1093/bioinformatics/btae505 (PMC11341121; doi:10.1093/bioinformatics/btae505)
Supplement: btae505_Supplementary_Data [file btae505_supplementary_data.pdf]

# Variance of the multivariate Mendelian randomization causal effects using the Delta method

Kaido Lepik

September 23, 2018

## Multivariate MR standard error

Consider the multivariate causal effect estimate

$$\hat{\mathbf{b}} = (\hat{\Gamma}' C^{-1} \hat{\Gamma})^{-1} \hat{\Gamma}' C^{-1} \hat{\gamma},$$

where $\hat{\Gamma}$ is an $M \times K$ matrix of standardized eQTL effect sizes, $C$ is an $M \times M$ LD-matrix between SNPs, and $\hat{\gamma}$ is a vector of standardized trait effect sizes of length $M$.

This is a function $f : \mathbb{R}^{M(K+1)} \to \mathbb{R}^K$ where $K$ is the number of risk factors and M is the number of SNPs. Define the following:

$$\boldsymbol{\beta} = \left(\begin{array}{c} \beta_{X_{11}} \\ \beta_{X_{21}} \\ \vdots \\ \beta_{X_{M1}} \\ \hline \beta_{X_{12}} \\ \beta_{X_{22}} \\ \vdots \\ \beta_{X_{M2}} \\ \hline \vdots \\ \hline \beta_{X_{1K}} \\ \beta_{X_{2K}} \\ \vdots \\ \beta_{X_{MK}} \\ \hline \beta_{Y_1} \\ \beta_{Y_2} \\ \vdots \\ \beta_{Y_M} \end{array}\right), \quad \boldsymbol{\sigma} = \left(\begin{array}{c} \sigma_{X_{11}} \\ \sigma_{X_{21}} \\ \vdots \\ \sigma_{X_{M1}} \\ \hline \sigma_{X_{12}} \\ \sigma_{X_{22}} \\ \vdots \\ \sigma_{X_{M2}} \\ \hline \vdots \\ \hline \sigma_{X_{1K}} \\ \sigma_{X_{2K}} \\ \vdots \\ \sigma_{X_{MK}} \\ \hline \sigma_{Y_1} \\ \sigma_{Y_2} \\ \vdots \\ \sigma_{Y_M} \end{array}\right), \quad \boldsymbol{\mu} = \left(\begin{array}{c} \mu_{X_{11}} \\ \mu_{X_{21}} \\ \vdots \\ \mu_{X_{M1}} \\ \hline \mu_{X_{12}} \\ \mu_{X_{22}} \\ \vdots \\ \mu_{X_{M2}} \\ \hline \vdots \\ \hline \mu_{X_{1K}} \\ \mu_{X_{2K}} \\ \vdots \\ \mu_{X_{MK}} \\ \hline \mu_{Y_1} \\ \mu_{Y_2} \\ \vdots \\ \mu_{Y_M} \end{array}\right)$$

where $\boldsymbol{\beta}$ is the vector of effect sizes ($\beta_{X_{mk}}$ corresponds to the effect of SNP $m$ on risk factor (gene expression) $k$ and $\beta_{Y_m}$ corresponds to the effect of SNP $m$ on the trait), $\boldsymbol{\sigma}$ is the vector of corresponding standard errors and $\boldsymbol{\mu}$ is the vector of corresponding mean effect sizes.

The Delta method gives us:

$$f(\boldsymbol{\beta}) \approx f(\boldsymbol{\mu}) + J(\boldsymbol{\mu})(\boldsymbol{\beta} - \boldsymbol{\mu})$$

where $J$ is a $K \times M(K+1)$ Jacobian matrix. Considering that $\mathrm{E}\boldsymbol{\beta} = \boldsymbol{\mu}$, we have

$$\mathrm{E}f(\boldsymbol{\beta}) \approx f(\boldsymbol{\mu}).$$

Therefore:

$$\mathrm{Var}[f(\boldsymbol{\beta})] = \mathrm{E}[(f(\boldsymbol{\beta}) - \mathrm{E}f(\boldsymbol{\beta}))(f(\boldsymbol{\beta}) - \mathrm{E}f(\boldsymbol{\beta}))']$$

$$\approx J(\boldsymbol{\mu})\mathrm{E}[(\boldsymbol{\beta}-\boldsymbol{\mu})(\boldsymbol{\beta}-\boldsymbol{\mu})']J'(\boldsymbol{\mu})$$
$$= J(\boldsymbol{\mu})\Sigma J'(\boldsymbol{\mu}), \tag{1}$$

where $\Sigma$ is the variance-covariance matrix of the effect size vector $\boldsymbol{\beta}$ and can be estimated using summary statistics and a reference dataset.

We need to take derivatives to find the $K \times M(K+1)$ Jacobian matrix

$$J = \left(\frac{df}{d\hat{\Gamma}}\middle|\frac{df}{d\hat{\gamma}}\right). \tag{2}$$

**First, we will find the derivative over $\hat{\Gamma}$.**

$$\frac{df}{d\hat{\Gamma}} = \frac{d(\hat{\Gamma}'C^{-1}\hat{\Gamma})^{-1}\hat{\Gamma}'C^{-1}\hat{\gamma}}{d\hat{\Gamma}} = \frac{d(\hat{\Gamma}'C^{-1}\hat{\Gamma})^{-1}\hat{\Gamma}'C^{-1}\hat{\gamma}}{d(\hat{\Gamma}'C^{-1}\hat{\Gamma})^{-1}\hat{\Gamma}'}\frac{d(\hat{\Gamma}'C^{-1}\hat{\Gamma})^{-1}\hat{\Gamma}'}{d\hat{\Gamma}} = \left((C^{-1}\hat{\gamma})' \otimes \mathbf{1}_K\right)\frac{d(\hat{\Gamma}'C^{-1}\hat{\Gamma})^{-1}\hat{\Gamma}'}{d\hat{\Gamma}}.$$

Let $Y = (\hat{\Gamma}'C^{-1}\hat{\Gamma})^{-1}$ and $Z = \hat{\Gamma}'$, then

$$\begin{aligned}\frac{d(\hat{\Gamma}'C^{-1}\hat{\Gamma})^{-1}\hat{\Gamma}'}{d\hat{\Gamma}} &= \left.\frac{d(\hat{\Gamma}'C^{-1}\hat{\Gamma})^{-1}Z}{d(\hat{\Gamma}'C^{-1}\hat{\Gamma})^{-1}}\right|_{Z=const}\frac{d(\hat{\Gamma}'C^{-1}\hat{\Gamma})^{-1}}{d\hat{\Gamma}} + \left.\frac{dY\hat{\Gamma}'}{d\hat{\Gamma}'}\right|_{Y=const}\frac{d\hat{\Gamma}'}{d\hat{\Gamma}} \\ &= \left(\hat{\Gamma} \otimes \mathbf{1}_K\right)\frac{d(\hat{\Gamma}'C^{-1}\hat{\Gamma})^{-1}}{d\hat{\Gamma}} + \left(\mathbf{1}_M \otimes (\hat{\Gamma}'C^{-1}\hat{\Gamma})^{-1}\right)P_{M,K},\end{aligned}$$

where $P_{M,K}$ is an $MK \times MK$ commutation matrix. We have

$$\frac{d(\hat{\Gamma}'C^{-1}\hat{\Gamma})^{-1}}{d\hat{\Gamma}} = \frac{d(\hat{\Gamma}'C^{-1}\hat{\Gamma})^{-1}}{d\hat{\Gamma}'C^{-1}\hat{\Gamma}}\frac{d\hat{\Gamma}'C^{-1}\hat{\Gamma}}{d\hat{\Gamma}} = \left(-(\hat{\Gamma}'C^{-1}\hat{\Gamma})^{-1} \otimes (\hat{\Gamma}'C^{-1}\hat{\Gamma})^{-1}\right)\frac{d\hat{\Gamma}'C^{-1}\hat{\Gamma}}{d\hat{\Gamma}}.$$

Let $Y = \hat{\Gamma}'$ and $Z = C^{-1}\hat{\Gamma}$, then

$$\begin{aligned}\frac{d\hat{\Gamma}'C^{-1}\hat{\Gamma}}{d\hat{\Gamma}} &= \left.\frac{d\hat{\Gamma}'Z}{d\hat{\Gamma}'}\right|_{Z=const}\frac{d\hat{\Gamma}'}{d\hat{\Gamma}} + \left.\frac{dYC^{-1}\hat{\Gamma}}{dC^{-1}\hat{\Gamma}}\right|_{Y=const}\frac{dC^{-1}\hat{\Gamma}}{d\hat{\Gamma}} \\ &= \left((C^{-1}\hat{\Gamma})' \otimes \mathbf{1}_K\right)P_{M,K} + (\mathbf{1}_K \otimes \hat{\Gamma}')(\mathbf{1}_K \otimes C^{-1}).\end{aligned}$$

Putting everything together and simplifying, we have

$$\frac{df}{d\hat{\Gamma}} = \frac{d(\hat{\Gamma}'C^{-1}\hat{\Gamma})^{-1}\hat{\Gamma}'C^{-1}\hat{\gamma}}{d\hat{\Gamma}} =$$
$$= \left((C^{-1}\hat{\gamma})' \otimes \mathbf{1}_K\right)\Big[(\hat{\Gamma} \otimes \mathbf{1}_K)\left(-(\hat{\Gamma}'C^{-1}\hat{\Gamma})^{-1} \otimes (\hat{\Gamma}'C^{-1}\hat{\Gamma})^{-1}\right)\left(((C^{-1}\hat{\Gamma})' \otimes \mathbf{1}_K)P_{M,K} + (\mathbf{1}_K \otimes \hat{\Gamma}')(\mathbf{1}_K \otimes C^{-1})\right) +$$
$$+ (\mathbf{1}_M \otimes (\hat{\Gamma}'C^{-1}\hat{\Gamma})^{-1})P_{M,K}\Big] =$$

$$= \left((C^{-1}\hat{\gamma})' \otimes \mathbf{1}_K\right)\Big[\left(-\hat{\Gamma}(\hat{\Gamma}'C^{-1}\hat{\Gamma})^{-1} \otimes (\hat{\Gamma}'C^{-1}\hat{\Gamma})^{-1}\right)\left(((C^{-1}\hat{\Gamma})' \otimes \mathbf{1}_K)P_{M,K} + (\mathbf{1}_K \otimes \hat{\Gamma}')(\mathbf{1}_K \otimes C^{-1})\right) +$$
$$+ \left(\mathbf{1}_M \otimes (\hat{\Gamma}'C^{-1}\hat{\Gamma})^{-1}\right)P_{M,K}\Big] =$$

$$= \left((C^{-1}\hat{\gamma})' \otimes \mathbf{1}_K\right)\left(-\hat{\Gamma}(\hat{\Gamma}'C^{-1}\hat{\Gamma})^{-1} \otimes (\hat{\Gamma}'C^{-1}\hat{\Gamma})^{-1}\right)\left(\left((C^{-1}\hat{\Gamma})' \otimes \mathbf{1}_K\right)P_{M,K}\right) +$$
$$+ \left((C^{-1}\hat{\gamma})' \otimes \mathbf{1}_K\right)\left(-\hat{\Gamma}(\hat{\Gamma}'C^{-1}\hat{\Gamma})^{-1} \otimes (\hat{\Gamma}'C^{-1}\hat{\Gamma})^{-1}\right)\left((\mathbf{1}_K \otimes (C^{-1}\hat{\Gamma})')\mathbf{1}_{MK}\right) +$$
$$+ \left((C^{-1}\hat{\gamma})' \otimes \mathbf{1}_K\right)(\mathbf{1}_M \otimes (\hat{\Gamma}'C^{-1}\hat{\Gamma})^{-1})P_{M,K} =$$

$$= \left(-\hat{\gamma}'C^{-1}\hat{\Gamma}(\hat{\Gamma}'C^{-1}\hat{\Gamma})^{-1} \otimes (\hat{\Gamma}'C^{-1}\hat{\Gamma})^{-1}\right)\left(((C^{-1}\hat{\Gamma})' \otimes \mathbf{1}_K)P_{M,K}\right) +$$

$$+\Big(-\hat\gamma' C^{-1}\hat\Gamma(\hat\Gamma' C^{-1}\hat\Gamma)^{-1} \otimes (\hat\Gamma' C^{-1}\hat\Gamma)^{-1}\Big)\Big((\mathbf{1}_K \otimes (C^{-1}\hat\Gamma)')\mathbf{1}_{MK}\Big)+$$
$$+\Big((C^{-1}\hat\gamma)' \otimes (\hat\Gamma' C^{-1}\hat\Gamma)^{-1}\Big) P_{M,K} =$$

$$= \Big(-\hat\gamma' C^{-1}\hat\Gamma(\hat\Gamma' C^{-1}\hat\Gamma)^{-1}\hat\Gamma' C^{-1} \otimes (\hat\Gamma' C^{-1}\hat\Gamma)^{-1}\Big) P_{M,K}+$$
$$+\Big(-\hat\gamma' C^{-1}\hat\Gamma(\hat\Gamma' C^{-1}\hat\Gamma)^{-1} \otimes (\hat\Gamma' C^{-1}\hat\Gamma)^{-1}\hat\Gamma' C^{-1}\Big)+$$
$$+\Big((C^{-1}\hat\gamma)' \otimes (\hat\Gamma' C^{-1}\hat\Gamma)^{-1}\Big) P_{M,K} =$$

$$= P_{K,1}\Big(-\hat\gamma' C^{-1}\hat\Gamma(\hat\Gamma' C^{-1}\hat\Gamma)^{-1}\hat\Gamma' C^{-1} \otimes (\hat\Gamma' C^{-1}\hat\Gamma)^{-1}\Big) P_{M,K}+$$
$$+\Big(-\hat\gamma' C^{-1}\hat\Gamma(\hat\Gamma' C^{-1}\hat\Gamma)^{-1} \otimes (\hat\Gamma' C^{-1}\hat\Gamma)^{-1}\hat\Gamma' C^{-1}\Big)+$$
$$+P_{K,1}\Big((\hat\gamma' C^{-1}) \otimes (\hat\Gamma' C^{-1}\hat\Gamma)^{-1}\Big) P_{M,K} =$$

$$= \Big((\hat\Gamma' C^{-1}\hat\Gamma)^{-1} \otimes \big(-\hat\gamma' C^{-1}\hat\Gamma(\hat\Gamma' C^{-1}\hat\Gamma)^{-1}\hat\Gamma' C^{-1}\big)\Big)+$$
$$+\Big(-\hat\gamma' C^{-1}\hat\Gamma(\hat\Gamma' C^{-1}\hat\Gamma)^{-1} \otimes (\hat\Gamma' C^{-1}\hat\Gamma)^{-1}\hat\Gamma' C^{-1}\Big)+$$
$$+\Big((\hat\Gamma' C^{-1}\hat\Gamma)^{-1} \otimes (\hat\gamma' C^{-1})\Big) =$$

$$= \Big((\hat\Gamma' C^{-1}\hat\Gamma)^{-1} \otimes \hat\gamma' C^{-1}\big(-\hat\Gamma(\hat\Gamma' C^{-1}\hat\Gamma)^{-1}\hat\Gamma' C^{-1} + \mathbf{1}_M\big)\Big)+$$
$$+\Big(-\hat\gamma' C^{-1}\hat\Gamma(\hat\Gamma' C^{-1}\hat\Gamma)^{-1} \otimes (\hat\Gamma' C^{-1}\hat\Gamma)^{-1}\hat\Gamma' C^{-1}\Big). \quad (3)$$

To get (3), we used the following properties:

- $(A \otimes B)(C \otimes D) = (AC) \otimes (BD)$,
- $A(B + C) = AB + AC$,
- $A(BC) = (AB)C$,
- $P_{K,1} = \mathbf{1}_K$,
- $P_{K,1}(A \otimes B)P_{M,K} = B \otimes A$, where $A : 1 \times M$ and $B : K \times K$,
- $A \otimes (B + C) = (A \otimes B) + (A \otimes C)$.

**Second, we will find the derivative over $\hat\gamma$.**

$$\frac{df}{d\hat\gamma} = \frac{d(\hat\Gamma' C^{-1}\hat\Gamma)^{-1}\hat\Gamma' C^{-1}\hat\gamma}{d\hat\gamma} = (\hat\Gamma' C^{-1}\hat\Gamma)^{-1}\hat\Gamma' C^{-1}. \quad (4)$$

We now have everything we need to estimate the multivariate MR standard error using the Delta method. We compared the SE obtained in this way to the SE obtained by Burgess' Bayesian MMR method in **Fig 1**.

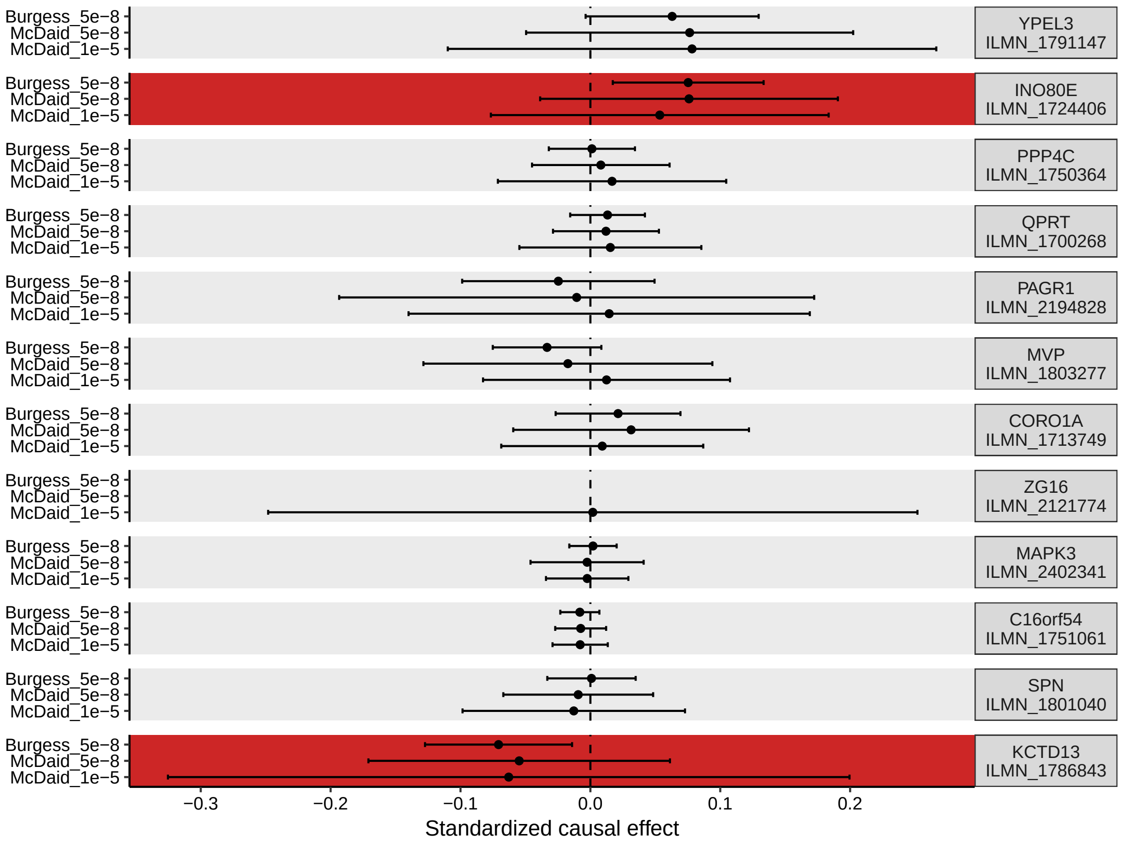


Figure 1: Standardized causal effect estimates for age at menarche with 95% confidence intervals using three different variants of multivariate Mendelian randomization. Standard errors for the McDaid method have been found using the above formulas. These are always bigger compared to the Burgess' Bayesian method in rjags. Furthermore (and somewhat interestingly), using more instruments (instrument selection threshold $10^{-5}$ compared to $5 \times 10^{-8}$) often results in bigger standard errors.
